# Editorial: Advances in circRNA research: disease associations and diagnostic innovations

**DOI:** 10.3389/fgene.2025.1759692

**Published:** 2025-12-19

**Authors:** Quan Zou, Mengting Niu, Xiaoqing Ru

**Affiliations:** 1 School of Artificial Intelligence, Shenzhen University of Information Technology, Shenzhen, China; 2 Institute of Fundamental and Frontier Sciences, University of Electronic Science and Technology of China, Chengdu, China; 3 Department of Integrative Biotechnology, College of Biotechnology and Bioengineering, Sungkyunkwan University, Suwon, Gyeonggi-do, Republic of Korea

**Keywords:** circRNA translation, circRNA-drug associations/sensitivity, circular RNA (circRNA), genetic susceptibility, graph-based modelling

## Introduction

Circular RNAs (circRNAs) are now recognised as important regulators of post-transcriptional gene expression (Gao et al., 2021; Liu et al., 2020; Liu et al., 2019; Zhao et al., 2020). Their covalently closed structure confers resistance to exonucleases, and many circRNAs are conserved across species, with expression patterns that are frequently tissue- and disease-specific (Ji et al., 2019; Wu et al., 2022). They are also detectable in plasma, serum, urine and other body fluids (Szabo and Salzman, 2016), making them attractive candidates for diagnostic and prognostic biomarkers and for monitoring treatment response (Ruan et al., 2019).

This editorial summarises six original and review articles in this Research Topic that, supported by multi-omics and computational methods, move beyond viewing circRNAs as isolated “differentially expressed molecules”. Together, these contributions place circRNAs within an integrated framework that links genetic risk, regulatory networks, drug response and potential therapeutic applications. In the sections below, we briefly discuss each article along these converging lines.

## From genome to endothelium: circRNAs as mediators of genetic risk

Genome-wide association studies (GWAS) have identified numerous loci for coronary artery disease (CAD), most of which lie in non-coding regions and are therefore difficult to link to specific cell types or pathways. Huang et al. integrate CAD GWAS signals with a circRNA-centred competing endogenous RNA (ceRNA) network and endothelial transcriptional programmes derived from intervention-based single-cell RNA sequencing data. In this framework, risk variants are mapped onto a multilayer network in which circRNAs connect GWAS loci to endothelial gene modules associated with extracellular matrix remodelling, cell adhesion and vascular homeostasis. By prioritising circRNAs that are both proximal to CAD-associated variants and strongly connected to these modules, they highlight circZNF609, circABCC1, circHERPUD2 and others as candidate mediators of genetic risk in the endothelium.

Rather than simply cataloguing GWAS loci or differentially expressed circRNAs, this work outlines a route from genetic susceptibility, through non-coding regulation, to endothelial pathways, and provides starting points for CRISPR-based perturbation, single-cell multi-omics and *in vivo* functional studies.

## From tumour-specific roles to pan-cancer hubs: circZFR across cancers

Nan et al. characterise circZFR as a “horizontal hub” across solid tumours. Drawing on studies in colorectal, hepatocellular and non-small cell lung cancer, among others, they summarise its expression patterns, clinical associations and major mechanistic links. CircZFR, generated by back-splicing of the ZFR gene and predominantly localised in the cytoplasm, is highly expressed in many tumour types and associated with advanced stage and poor prognosis. Mechanistically, it acts as a sponge for miRNAs that repress oncogenic targets and stabilises proteins such as BCLAF1 and HNRNPLL, thereby engaging canonical oncogenic pathways and promoting tumour cell proliferation, invasion, metastasis and resistance to cisplatin and other chemotherapeutic agents.

In some signalling contexts, circZFR appears to exert tumour-suppressive effects, underscoring the context dependence of its function. Overall, these findings suggest that some circRNAs act not only as local modulators in individual diseases but also as shared hubs across tumour types and treatment stages, thereby providing a template for the systematic identification of other high-value circRNAs.

## Towards drug response and intervention: from graph models to translation

Three articles extend circRNA research towards therapeutic relevance, covering circRNA-drug associations, circRNA-drug sensitivity and the translational capacity of circRNAs as diagnostic or therapeutic resources.

Li et al. (2025) focuses on circRNA-drug associations. On a circRNA-drug bipartite graph, local higher-order topology is encoded as geometric features within a graph neural network (GNN), helping the model distinguish edges in structured subnetworks from more random connections. On public datasets, G2CDA outperforms earlier approaches, and several top-ranked predicted pairs are supported by existing evidence, showing how geometric deep learning can prioritise circRNA targets and candidates for drug development or repurposing.

Wang et al. (2025) addresses circRNA-drug sensitivity. It constructs a heterogeneous network integrating circRNA and drug similarity information with circRNA-drug sensitivity data, applies graph contrastive learning to obtain representations robust to noise and missing links, and then uses multi-view GNNs to predict the sensitivity of circRNA-drug pairs. DMAGCL identifies candidate circRNA markers associated with the response to multiple anticancer agents and links circRNA networks more directly to treatment response.

Zhang et al. review circRNA translation and emphasise that some circRNAs are not strictly non-coding. They survey the molecular mechanisms, experimental assays and computational tools used to study circRNA-derived peptides and proteins, and argue that these translation products are relevant to tumourigenesis and cell fate control and could underpin new diagnostic markers and RNA- or peptide-based therapeutic strategies.

These three contributions support a change in perspective: circRNAs are moving from being viewed mainly as molecules influenced by drugs to being considered components that help determine drug response and, in some cases, substrates for therapeutic design.

## Hidden foundations: MSA post-processing as infrastructure for circRNA research

Zhai et al. review post-processing strategies for multiple sequence alignments (MSAs), organising meta-alignment together with horizontal, vertical and hybrid realignment, and showing how such refinement can improve alignment quality and the robustness of downstream analyses without changing the underlying alignment engine. Although circRNAs are not the focus of their article, circRNA studies depend critically on reliable MSAs: cross-species conservation of circRNAs and their ORF or IRES segments, miRNA and RNA-binding protein recognition sites, and comparative evidence for translational potential all build on trustworthy alignments. These general methods therefore constitute core infrastructure for sequence- and network-level analyses, including work on circRNA translation and graph-based modelling.

## Conclusion and outlook: from association landscapes to clinical pathways

The six articles collectively delineate several main lines of circRNA research: one linking genetic susceptibility to cell type-specific pathways, another spanning tumour types and drug response, and a third extending to translation products and methodological infrastructure. Future advances are likely to arise at the intersections of these lines, for example, by systematically integrating GWAS, single-cell and epigenomic data in large multimodal cohorts and using graph-based models together with translation-prediction tools to identify and validate circRNAs with genuine diagnostic or therapeutic value. This Research Topic does not aim to provide definitive answers, but rather to offer a set of mutually reinforcing “pieces of the puzzle” that may guide more systematic and translational work at the intersection of circRNA biology, disease research and computational methodology.
